# Pancreatic serous cystic neoplasm mimicking intraductal papillary mucinous neoplasm: Two case reports and literature review

**DOI:** 10.1097/MD.0000000000032820

**Published:** 2023-02-03

**Authors:** Mihyeon Park, Jisun Lee, Yook Kim, Kyung Sik Yi, Bum Sang Cho, Chi-Hoon Choi, Kil Sun Park

**Affiliations:** a Department of Radiology, Chungbuk National University Hospital, Cheongju, Republic of Korea; b Department of Radiology, College of Medicine, Chungbuk National University, Chungbuk National University Hospital, Cheongju, Republic of Korea.

**Keywords:** algorithms, and serous, case report, cystic, mucinous, neoplasms, pancreatic cyst, pancreatic intraductal neoplasms, risk assessment

## Abstract

Serous cystic neoplasms (SCNs) are treated as benign lesions. It is widely known that SCN rarely have a connection with the main pancreatic duct (MPD), which helps differentiate them from other cystic lesions, such as intraductal papillary mucinous neoplasm (IPMN). However, very rare cases where the SCN appears connected to the MPD cause diagnostic confusion.

**Patient concerns:**

We present 2 patients with SCN (1 male, 54, and 1 female, 42). Both patients were asymptomatic, without abnormal laboratory results.

**Diagnosis:**

In both cases, abdominopelvic computed tomography and pancreatic magnetic resonance imaging scans revealed a multilobulated cystic lesion in communication with the MPD. Since the size of each patient’s lesion was >3 cm and there was connectivity with the MPD, it was strongly suspected to be a branch duct-type IPMN with worrisome features rather than SCN and surgical intervention was considered.

**Interventions:**

Both neoplasms were misdiagnosed as IPMN due to appearing connected with the MPD on radiologic imaging. Surgery was performed.

**Outcomes:**

A final diagnosis of microcystic serous cystadenoma of the pancreas without connectivity of MPD was confirmed in both patients.

**Lessons:**

An unnecessary surgery was performed due to atypical radiologic features in which the pancreatic duct seems to be connected to the pancreatic cystic lesion on magnetic resonance imaging, leading to misdiagnosis of SCN as IPMN. Particular attention should be paid to interpretation of clinicoradiologic findings of pancreatic cystic lesions, especially to the decision of surgical intervention. Also, awareness of presence of the atypical radiologic features of SCN may broaden the knowledge base of radiologists.

**Lessons:**

An unnecessary surgery was performed due to atypical radiologic features in which the pancreatic duct seems to be connected to the pancreatic cystic lesion on magnetic resonance imaging, leading to misdiagnosis of SCN as IPMN. Particular attention should be paid to interpretation of clinicoradiologic findings of pancreatic cystic lesions, especially to the decision of surgical intervention. Also, awareness of presence of the atypical radiologic features of SCN may broaden the knowledge base of radiologists.

## 1. Introduction

Serous cystic neoplasms (SCN) of the pancreas are benign, although there are a few cases of malignant transformation.^[1]^ The radiologic features of SCN have been described as microcystic, macrocystic (or oligocystic), mixed microcystic and macrocystic, and solid types.^[2]^ Representatively, microcystic SCNs are multiloculated cystic lesions, and a central scar can be present.^[2]^ It is widely known that SCN rarely have a connection with the main pancreatic duct (MPD), a characteristic that may help differentiate them from other cystic lesions such as intraductal papillary mucinous neoplasm (IPMN).^[3]^ Although, there are some cases in which the SCN appears to be connected to the MPD, it is not commonly recognized and can lead to misdiagnosis.^[3–6]^

Herein, we report 2 atypical cases of SCN of the pancreas that mimicked IPMN owing to the imaging features of communication with the MPD. We reviewed several other cases in which SCN was confused with IPMN and underwent surgery, as the cystic lesion of the pancreas appeared to be associated with the MPD in imaging findings. We also discuss the reasons for such an appearance and how unnecessary surgery can be reduced by applying the algorithmic approach. The case report was produced in compliance with the EQUATOR (Enhancing the QUAlity and Transparency Of health Research) Network CARE guidelines.^[7]^

### 1.1. Case 1

In March 2021, a 54-year-old man underwent investigation for the staging of skin lymphoma in our hospital. During the metastatic examination, a multilobulated cystic mass in the pancreatic body was incidentally detected on abdominopelvic computed tomography (APCT) scans. The patient did not complain of any specific symptoms and the patients’ vital signs and physical examination results were unremarkable at the time of examination. The patient had no remarkable medical history other than previous pulmonary tuberculosis and don’t have a family history of cancer. Laboratory examination results in patient were within the normal range, including serum carbohydrate antigen 19-9 and carcinoembryonic antigen (CEA) levels.

**Figure 1.**
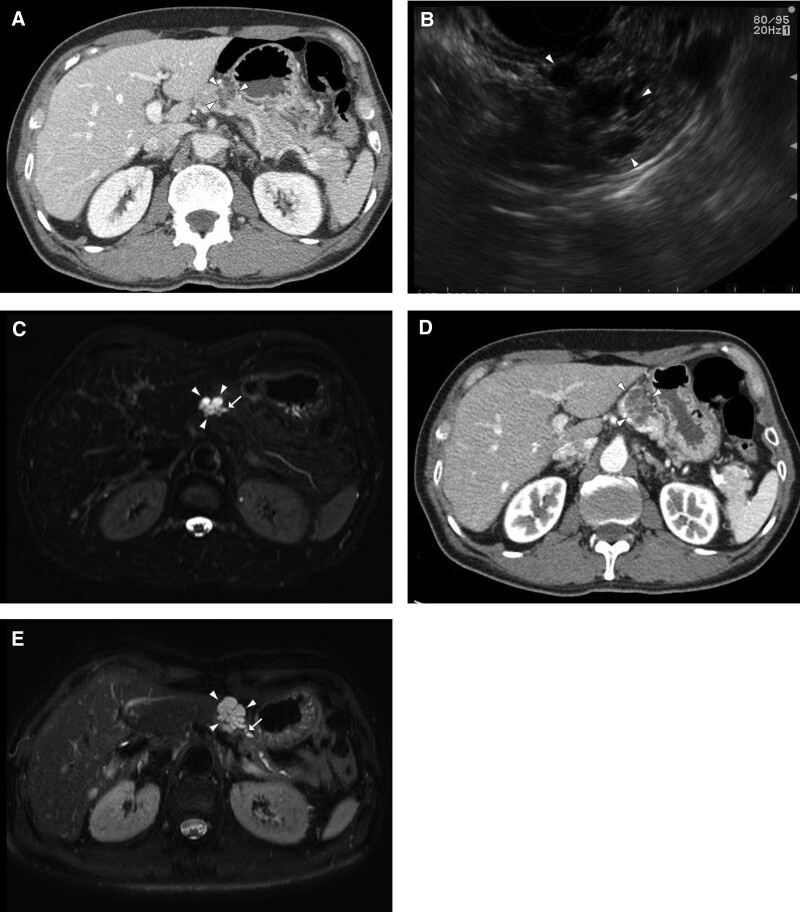
(A) Initial abdominopelvic computed tomography (APCT) revealed a roughly 2.2 cm sized irregular multilobulated low-density lesion (arrowheads) in the pancreatic body. (B) Endoscopic ultrasonography showed macrocystic (arrowheads), and honeycomb pattern with central sunburst-like appearance. (C) Pancreas magnetic resonance imaging (MRI) revealed that the main pancreatic duct (MPD) (D, arrow) appeared to be communicated with multilobulated cystic lesion (arrowheads) in pancreas. (D) Follow-up APCT showed that the pancreatic cystic lesion (arrowheads) gradually increased in size by 1.4 cm in the 4 years. (E) Additional pancreas MRI revealed that dilatation of the distal MPD (arrow) and the connection of MPD with the pancreatic cystic lesion (arrowheads) were more pronounced.

Initial APCT showed an irregular, multilobulated, low-density lesion of approximately 2.2 cm in the pancreatic body (Fig. 1A).

Endoscopic ultrasonography (EUS) revealed a macrocystic and honeycombing pattern with a central sunburst-like appearance in the pancreatic lesion, and there was no significant dilatation of the MPD (Fig. 1B).

However, a follow-up pancreatic magnetic resonance imaging (MRI) scanning was performed, which revealed that the distal MPD was dilated to 0.4 cm and appeared to be communicating with multilobulated cystic lesions in the pancreas (Fig. 1C), making IPMN the most likely diagnosis rather than SCN.

There was no evidence of high-risk or worrisome features for pancreatic IPMN on MRI scans, and an annual follow-up with APCT was performed for pancreatic cystic lesion. The pancreatic cystic lesion gradually increased in size on annual APCT scans, and the maximum diameter of the pancreatic cystic lesion increased by 0.4 cm in the first 2 years and 1.0 cm in the next 2 years (Fig. 1D). An additional pancreatic MRI scanning was performed, which showed that dilatation of the distal MPD – up to 3.5 mm – and the connection of MPD with the pancreatic cystic lesion – maximum diameter of 3.6 cm – were more pronounced (Fig. 1E).

Both APCT and pancreatic MRI findings and follow-up imaging findings were suggestive of IPMN of the pancreas because of connectivity with the MPD rather than SCN, as seen in the initial EUS findings. However, as the size had increased by 1.0 cm in 2 years, if it was indeed an IPMN, according to the 2017 Fukuoka guidelines,^[8]^ it could be considered to have a worrisome feature.

The patient underwent a distal pancreatectomy. During the operation, it was noted that a lesion with a diameter of approximately 4 cm was present in the pancreatic body. Subsequently, laparoscopic resection of the body and tail of the pancreas was performed.

**Figure 2.**
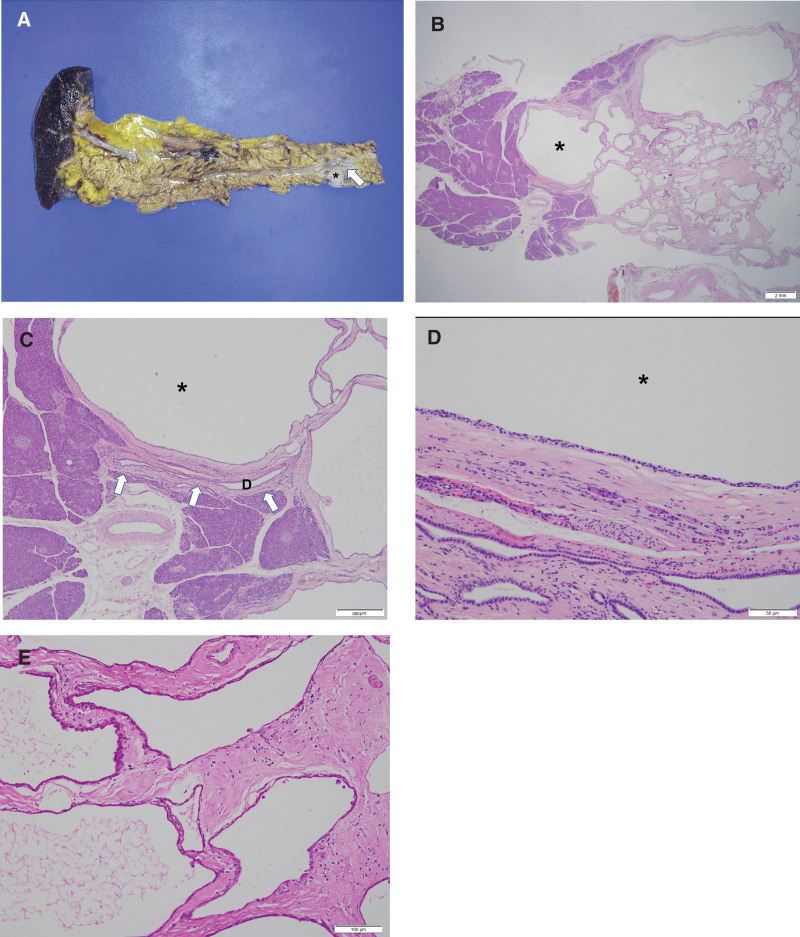
(A) The macroscopic view of the resected pancreas shows a well-defined multiloculated cystic mass (asterisk), measuring 3.2 × 2.0 × 1.4 cm^3^, with the main pancreatic duct (MPD) (arrow) running close to the lesion. (B) Multiloculated microcystic lesion (asterisk) was identified in the pancreatic parenchyma (hematoxylin and eosin, scale bar 2mm). (C) The MPD (D, arrow) is located very close to, and compressed by, the cystic lesion (asterisk), but there is no communication between them (hematoxylin and eosin, scale bar 500 µm). (D) Multilocular cyst (asterisk) lined by cuboidal epithelial cells contains clear cytoplasm and hyaline stroma (hematoxylin and eosin, scale bar 50 µm). (E) On immunohistochemical analysis (scale bar 100 µm), the lining cells appear positive for Periodic acid–Schiff (PAS), indicating glycogen-rich cytoplasm.

The macroscopic view of the resected pancreas contained a well-defined multiloculated cystic mass (asterisk) measuring 3.2 × 2.0 × 1.4 cm^3^, and the MPD (arrow) running close to the lesion was identified (Fig. 2A).

Histopathological examination of the pancreatic cystic lesion (asterisk) revealed a multiloculated microcystic lesion in the pancreatic parenchyma (hematoxylin and eosin, ×12.5) (Fig. 2B). The MPD (D, arrow) was located very close to the cystic lesion (asterisk) and was compressed by the cystic lesion, but there was no communication between them (hematoxylin and eosin, ×40) (Fig. 2C). Multilocular cysts (asterisk) were lined with cuboidal epithelial cells and contained clear cytoplasm and hyaline stroma (hematoxylin and eosin, ×200) (Fig. 2D). On immunohistochemical analysis (×200), the lining cells stained positive for periodic acid–Schiff (PAS), which is indicative of a glycogen-rich cytoplasm (Fig. 2E). A final diagnosis of microcystic serous cystadenoma of the pancreas was established.

The patient’s clinical course was uneventful, and he was discharged 2 weeks post-surgery. The patient had no complications for up to 3 months postoperatively.

### 1.2. Case 2

In March 2021, a 42-year-old woman presented to our hospital with incidental abnormal findings on abdominal US scans during a medical checkup. Abdominal US and APCT revealed a multilobulated cystic mass in the pancreatic body. The patient did not complain of any specific symptoms and the patients’ vital signs and physical examination results were unremarkable at the time of examination. The patient had a medical history of multiple sclerosis, for which she was taking carbamazepine, and asthma, for which she was taking daily inhalants. The patient does not have a family history of cancer. Laboratory examination results in patient were within the normal range, including serum carbohydrate antigen 19-9 and CEA levels.

**Figure 3.**
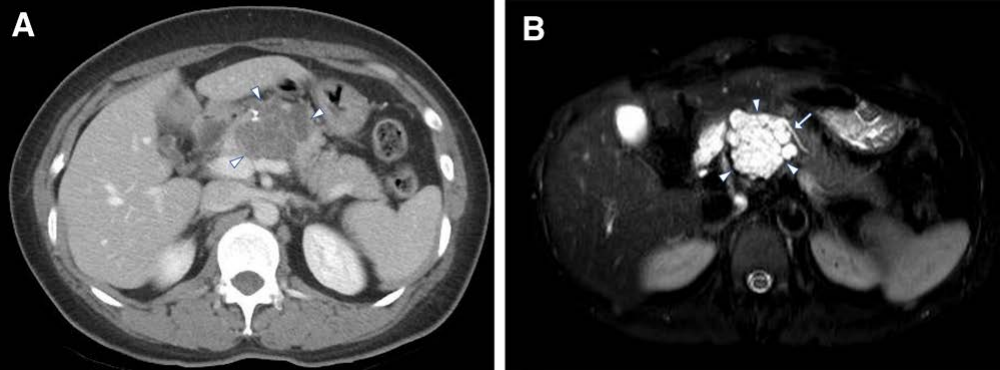
(A) Initial abdominopelvic computed tomography revealed a multilobulated low-density lesion (arrowheads) of approximately 5 cm with multiple enhancing septations and calcifications on pancreatic body. (B) Pancreas magnetic resonance imaging revealed a multiloculated cystic lesion (arrowheads) at the body of pancreas which appeared to be communicated with the main pancreatic duct (arrow).

Initial APCT revealed an approximately 5 cm multilobulated low-density lesion with multiple enhancing septations and calcifications in the pancreatic body (Fig. 3A). Pancreatic MRI revealed a multiloculated cystic lesion in the body of the pancreas that communicated with the MPD (Fig. 3B).

As there was a radiographically clear connection between the pancreatic cystic mass and MPD, and the size of the lesion was >3 cm, it was strongly suspected to be a branch duct-type IPMN with worrisome features rather than SCN.

A median pancreatectomy was performed. During the operation, it was noted that a cystic mass was present in the pancreatic body with an abutment to the splenic vein. Subsequently, resection of the body of the pancreas and the splenic vein was performed with pancreaticojejunostomy.

**Figure 4.**
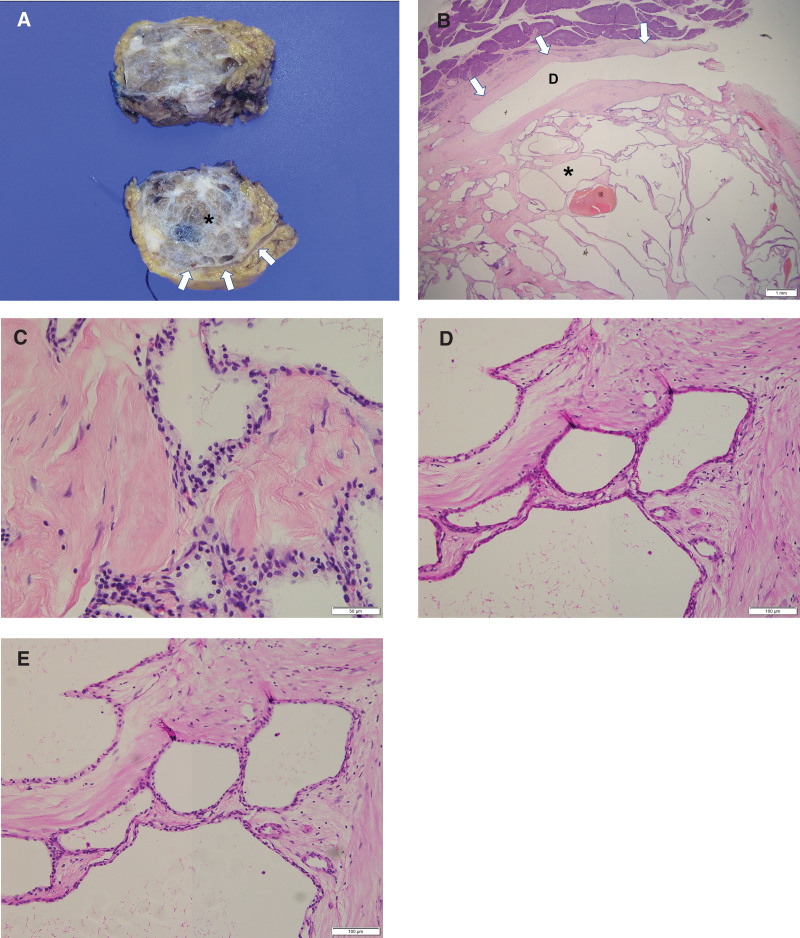
(A) The macroscopic view of the resected pancreas shows a well-defined multiloculated cystic mass (asterisk), measuring 4.1 × 4.0 × 3.1 cm^3^, and the main pancreatic duct (MPD) (arrows) runs close to the lesion. (B) Multiloculated microcystic lesion identified in the pancreatic parenchyma and the MPD (D, arrows) is close to the cystic lesion, nevertheless, not involved with the cystic lesion (asterisk) (hematoxylin and eosin, scale bar 1mm). (C) Multilocular cyst (asterisk) is lined by cuboidal epithelial cells and contains clear cytoplasm and hyaline stroma (hematoxylin and eosin, scale bar 50 µm). (D) On immunohistochemical analysis (scale bar 100 µm), the lining cells appear positive for Periodic acid–Schiff (PAS) and(E) negative for PAS with diastase digestion, which are indicative of glycogen-rich and diastase-digestible cytoplasm.

The macroscopic view of the resected pancreas contained a well-defined multiloculated cystic mass (asterisk), measuring 4.1 × 4.0 × 3.1 cm^3^, and the MPD running close to the lesion was identified (arrow) (Fig. 4A).

Histopathological examination of the pancreatic cystic lesion revealed a multiloculated microcystic lesion (asterisk) in the pancreatic parenchyma. The MPD (D, arrow) was close to the cystic lesion but did not involve it (hematoxylin and eosin, ×10), as in the macroscopic view (Fig. 4B). The multilocular cyst was lined with cuboidal epithelial cells and contained clear cytoplasm and hyaline stroma (hematoxylin and eosin, ×400) (Fig. 4C). On immunohistochemical analysis (×200), the lining cells stained positive for PAS and negative for PAS with diastase digestion, indicative of glycogen-rich and diastase-digestible cytoplasm (Fig. 4D and E). A final diagnosis of microcystic serous cystadenoma of the pancreas without a connection to the MPD was confirmed.

The patient recovered well, without any postoperative complications, and was discharged 10 days after surgery. The patient was followed up for 3 months, and no abnormal findings were observed on CT examinations.

## 2. Discussion

SCN is a benign entity.^[9]^ Despite a few reports of serous cystadenocarcinomas, typical SCNs should be treated as benign lesions, as the possibility of malignant transformation is negligible.^[1]^

The morphological varieties of SCN include microcystic, macrocystic (or oligocystic), mixed microcystic and macrocystic, and solid. Microcystic SCNs are composed of multiple small cysts with a honeycomb appearance. Central calcification or scarring can be present in the SCN. Macrocystic (or oligocystic) SCNs are composed of fewer, larger cysts.^[2]^

IPMNs can be classified as low-grade dysplasia, high-grade dysplasia, or associated invasive carcinoma.^[10]^ Because of the variable risk of malignancy, it is important to predict the malignant potential of pancreatic IPMNs using imaging findings, as management differs based on malignancy risk. International consensus guidelines were established in 2006^[11]^ to aid in the management of pancreatic IPMN, and revised versions were established in 2012^[12]^ and 2017,^[8]^ proposing high-risk stigmata and worrisome features, respectively, for determining the malignant potential of IPMNs.

High-risk stigmata and worrisome features were defined to stratify the risk of malignancy in IPMN and consider the frequency of surveillance or resection. High-risk stigmata include enhancing mural nodules within the cyst measuring ≥5 mm, MPD diameter measuring ≥10 mm, and obstructive jaundice. Worrisome features include enhancing mural nodules of <5 mm, a cyst growth rate >5 mm per 2 years, and elevated levels of serum CA19-9.^[8]^

Generally, branch duct-type IPMNs are typically distinguished by their tendency to communicate with the pancreatic duct system, unlike SCN.^[3]^ IPMN is a tumor that arises from the epithelial cells of the pancreatic duct. Excessive secretion of mucus blocks the pancreatic duct, resulting in dilatation of the pancreatic duct.^[13]^ In cases of branch duct-type IPMN, involvement is limited to segmental ducts, resulting in a solitary cystic mass without definite dilatation of the MPD with multiple intercommunicating cysts. Today, the gold standard for the study of the ductal system is MRI with magnetic resonance cholangiopancreatography, since it is especially sensitive in providing unique information on the relationship of the cystic lesions with the ductal system. T2-weighted images show a high-signal intensity in the dilated duct and effectively demonstrate the complex cystic mass connected to the pancreatic duct. Additionally, MRI may occasionally delineate mural nodules, finding high-risk stigmata or worrisome features.^[1]^

Nevertheless, there are various unusual features of SCN found in imaging studies that may lead to diagnostic difficulties. The main atypical manifestations include SCN with interval growth, SCN communication with the MPD, and giant tumors causing ductal dilatation.^[5]^ These characteristics may confuse SCN with IPMN, which may change treatment options, as in our cases.

**Figure 5.**
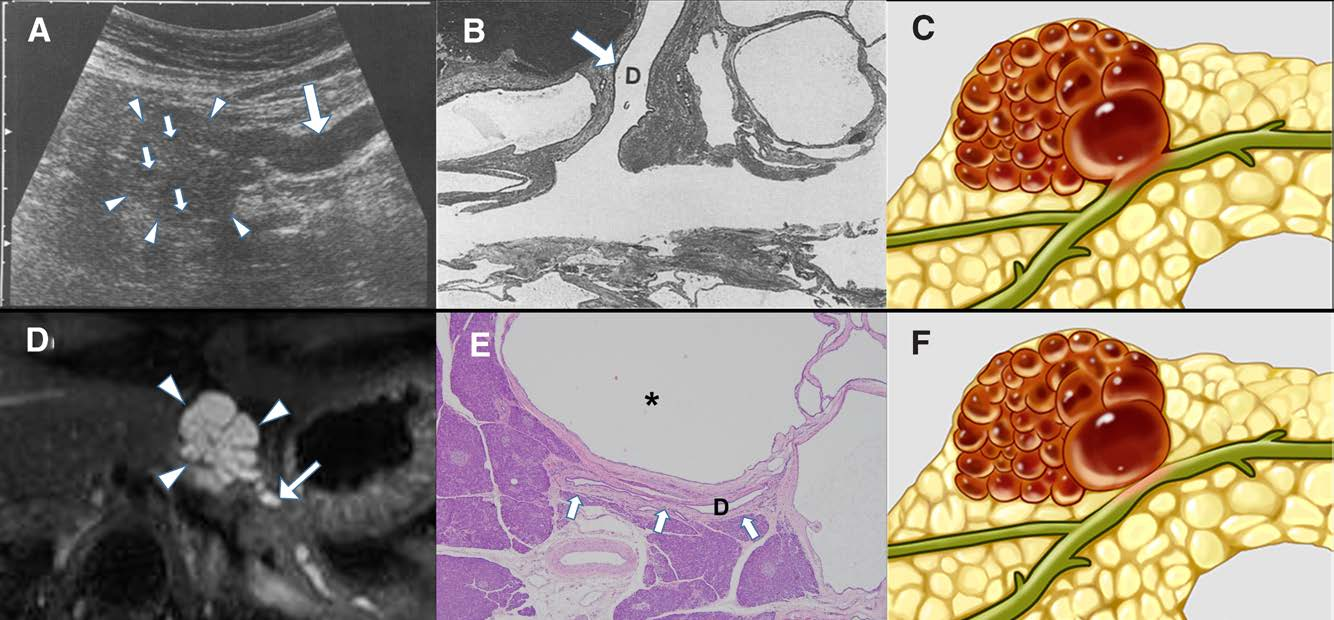
Schematic diagram of the correlation of histopathologic findings with radiologic findings in the case of Furukawa et al (1996)^[3]^ and our second case. Serous cystadenoma communicated with the main pancreatic duct (MPD); transabdominal ultrasonography demonstrated a lobulated hypoechoic mass (arrowheads) with small cystic components (small arrows). (A) The distal portion of the MPD (large arrow) is dilated. (B) On histopathologic examination, multiple small cysts lined with a layer of flat epithelium and pancreatic duct (D, large arrow) communicating with the cystic lesion were identified. (C) Schematic description of communication between the pancreatic cystic lesion and pancreatic duct possibly due to erosive change and secondary fistula formation of the pancreatic duct. (D) Serous cystadenoma without connection with the MPD but with external compression of the MPD mimicking intraductal papillary neoplasm on imaging findings; Magnetic resonance imaging of the pancreas revealed a multiloculated cystic lesion (arrowheads) at the body of the pancreas that appeared to be communicating with the MPD (arrow). (E) The pancreatic duct (D, arrows) was very close to, and compressed by, the cystic lesion (asterisk) but not involved with the cystic lesion. (F) Schematic description of the compressed morphology of the pancreatic duct by the pancreatic cystic lesion, resulting in radiologic misdiagnosis of a true connection with the pancreatic duct.

In our 2 cases, the lesions had an appearance similar to that of SCN owing to their lobulating contour and honeycombing.^[13]^ However, since connectivity with the MPD is a very uncommon feature in SCN that it is rarely considered a possibility in clinical practice,^[1,5]^ the lesion was presumed to be an IPMN. According to a 2012 Japanese nationwide survey, pancreatic duct communication with SCN exists, despite low rates of 9% of the total, 8% of the microcystic type, 0% of the mixed-type and solid-type, and 15% of the macrocystic type.^[14]^ According to our PubMed keyword survey, there are a few case reports of pancreatic cystic lesions in communication with the MPD in imaging studies that were presumed to be IPMN, but pathology revealed a diagnosis of SCN (Table 1). Considering the reasons for the confusion in diagnosis, cystic lesions could compress the MPD extrinsically, causing MPD dilatation. This could lead to erosion of the pancreatic duct and cause recurrent pancreatitis, resulting in the formation of a secondary fistula and inducing the possibility of a true connection to the MPD.^[3,4,6]^ However, in some cases, the histopathologic exams revealed no connectivity of the cystic lesions with the MPD; thus, the appearance of the external compressed and subsequently dilated MPD may have raised suspicion of the connectivity with the MPD morphologically on images, as in our cases.^[5]^ The former case, SCN with true connection to MPD with the erosion of pancreatic duct, corresponds to the case of Furukawa et al (1996),^[4]^ and the case of SCN without MPD connection but with external MPD compression corresponds to our 2 cases. These observations can be expressed in a schematic illustration based on histopathologic findings compared to radiologic findings (Fig. 5).

**Table 1:** Summary of clinical characteristics and radiologic features of previously reported cases of serous cystic neoplasm mimicking intraductal papillary mucinous neoplasm.

Reference	Age/Sex	Clinical manifestations	Laboratory findings	CT findings	MR findings	ERCP findings	EUS findings	FNA findings	Treatment	Pathologic findings	Follow-up
Furukawa et al^[3]^	76/M	Vague abdominal pain	NA	Multicystic enhanced mass composed of a spongy meshwork with central scar	NA	Communication between pancreatic duct and the cystic cavity	NA	NA	Pancreatico-duodenectomy	Serous cystadenoma communicated with the pancreatic duct	NA
Hashimoto et al^[24]^	42/F	Incidental finding	No elevation of levels of amylase, lipase, or tumor markers	Multiple cystic tumors, the wall of which had no enhancing solid component	Cystic tumor of low intensity on T1-weighted images and high intensity on T2-weighted images	The deviation of the MPD and inflow of the contrast material into the cystic part of the tumor	NA	NA	Pylorus-preserving pan creaticoduodenectomy	Serous cystadenoma communicated with the pancreatic duct	Doing well 12 mo after surgery
JaeHong Jung et al^[5]^	44/F	Right upper quadrant pain and radiating pain	Elevation of serum amylase and lipase levels No elevation of CA 19-9 level	Multilocular cystic mass in the body of the pancreas with dilated pancreatic duct	NA	The inflow of the contrast material into the cystic part of the tumor	NA	NA	Distal pancreatectomy	Serous cystadenoma communicated with the pancreatic duct	NA
Stephanie Truant et al^[4]^	66/F	Transient epigastric pain	No elevation of CEA and CA 19-9 levels	Well-defined, multiloculated, cystic mass	Upstream dilatation of the main pancreatic duct	Cystic dilatation of pancreatic branches communicating with the dilated MPD	Microcystic pattern concluded in serous cystadenoma	CEA (0.2 ng/mL)	Distal pancreatectomy	Serous cystadenoma with external compression of the MPD	Doing well 6 mo after surgery
Berman et al^[2]^	65/F	Right upper quadrant pain	NA	Cystic mass in the head and neck of the pancreas	Side-branch communication between the cystic lesion and the pancreatic duct	NA	NA	NA	Pancreatico-duodenectomy	Serous cystadenoma in direct communication with the pancreatic duct	Doing well 8 mo after surgery
Matsubayashi et al^[22]^	59/F	Incidental finding	Mild elevation of CEA level (7.5 ng/mL)	Multilocular cyst with partially thickened septum	Cystic lesion with dilated upstream MPD	Compressed, atrophic Wirsung duct and stenotic Santorini duct, connection with the multilocular cyst at the pancreatic head	Macroscopic multilocular cyst with honeycomb- like components and irregularly dilated MPD with lobularity and high echoic foci	High level of amylase (347000 U/L), CA 19-9 (30796 U/mL), but low level of CEA (4.5 ng/mL)	Pancreatico-duodenectomy	Serous cystadenoma communicated with the pancreatic duct	Doing well 3 yr after surgery
Case 1	54/M	Incidental finding	No elevation of CEA and CA 19-9 levels	Multiloculated, cystic mass in the body of the pancreas	Communication between the cystic lesion and the pancreatic duct	NA	Microcystic pattern with central scar in pancreatic lesion	NA	Distal pancreatectomy	Serous cystadenoma without connection with the MPD	Doing well 6 mo after surgery
Case 2	42/F	Incidental finding	No elevation of CEA and CA 19-9 levels	Multiloculated, cystic mass in the body of the pancreas	Communication between the cystic lesion and the pancreatic duct	NA	NA	NA	Median pancreatectomy	Serous cystadenoma without connection with the MPD	Doing well 6 mo after surgery

CA 19-9, carbohydrate antigen 19-9, CEA = carcinoembryonic antigen, CT = computed tomography, ERCP = endoscopic retrograde cholangiopancreatography, EUS = endoscopic ultrasonography, FNA = fine-needle aspiration, IPMN = intraductal papillary mucinous neoplasm, MPD = main pancreatic duct, MR = magnetic resonance, NA = not available, SCN = serous cystic neoplasm.

Regarding cases of diagnostic uncertainty, as in our cases, it is important to identify those lesions in need of resection and those that may be safely monitored with risk stratification. The algorithmic approach has been proposed by several groups for the diagnosis and management of incidental pancreatic cystic lesions. Although there were differences in the details, the direction of the large flow was generally consistent.^[5,8,9,15–20]^ According to the guidelines,^[8,9,20]^ the indications for surgical resection of pancreatic cystic lesion were mostly overlapping, as summarized in Table 2.^[2]^

**Table 2:** Indications for endoscopic ultrasonography-fine-needle aspiration of pancreatic cysts by different guidelines.

Guidelines	AGA (2015)^[20]^	IAP (2017)^[8]^	European (2018)^[9]^
	At least 2 of the following features:	• Growth rate ≥ 5 mm/2 yr	• If the results are expected to change clinical management
	• Cyst diameter > 30 mm	• Serum CA 19-9 level↑	
		• PD dilatation 5–9 mm	
	• PD dilatation	• Cyst diameter ≥ 30 mm	
			• If there are clinical or radiological features of concern during the initial investigation or surveillance
		• Enhancing mural nodule < 5 mm	
	• Solid nodule		
		• Lymphadenopathy	
		• Abrupt change of PD diameter and distal pancreatic atrophy	
		• Thickened or enhancing wall of cyst	

AGA = American Gastroenterological Association Institute guideline on the diagnosis and management of asymptomatic neoplastic pancreatic cysts, CA 19-9, carbohydrate antigen 19-9, European = European evidence-based guidelines on pancreatic cystic neoplasms, EUS = endoscopic ultrasonography, FNA = fine-needle aspiration, IAP = Revisions of International Consensus Fukuoka Guidelines for the Management of intraductal papillary mucinous neoplasm of the pancreas, PD = pancreatic duct.

The indications for EUS-fine needle aspiration (FNA) in IPMN are radiological features of concern in other cross-sectional images (such as worrisome features), and the results of EUS-FNA can help to classify risks more accurately and evaluate considerations for surgery. The indications for EUS-FNA in IPMN showed some differences between the various groups, as summarized in Table 3.^[2,8,9,20]^

**Table 3:** Indications for surgical resection of intraductal papillary mucinous neoplasm by different guidelines.

Guidelines	AGA (2015)^[20]^	IAP (2017)^[8]^	European (2018)^[9]^
Absolute indications	• PD dilatation ≥ 5 mm	• Positive or suspicious cytology results for malignancy	• Positive cytology results for malignancy or high-grade dysplasia
			• Enhancing mural nodule ≥ 5 mm
		• Enhancing mural nodule ≥ 5 mm	
	• Solid portion or positive cytology results for malignancy		
		• PD dilatation ≥ 10 mm	
		• Jaundice related with IPMN	• PD dilatation ≥ 10 mm
			• Solid portion
			• Jaundice related with IPMN
Relative indications	-	• Growth rate ≥ 5 mm/2 yr	• Growth rate ≥ 5 mm/2 yr
		• Serum CA 19-9 level↑	
			• Serum CA 19–9 level↑
		• PD dilatation 5–9 mm	
		• Cyst diameter ≥ 30 mm	• PD dilatation 5–9.9 mm
		• Enhancing mural nodule < 5 mm	
			• Cyst diameter ≥ 40 mm
		• Lymphadenopathy	
		• Abrupt change of PD diameter and distal pancreatic atrophy	• Enhancing mural nodule < 5 mm
		• Thickened or enhancing wall of cyst	• New-onset diabetes mellitus
		• Acute pancreatitis caused by IPMN	• Acute pancreatitis caused by IPMN

AGA = American Gastroenterological Association Institute Guideline on the Diagnosis and Management of Asymptomatic Neoplastic Pancreatic Cysts, CA 19-9, carbohydrate antigen 19-9, European = European evidence-based guidelines on pancreatic cystic neoplasms, IAP = Revisions of International Consensus Fukuoka Guidelines for the Management of IPMN of the pancreas, IPMN = intraductal papillary mucinous neoplasm, PD = pancreatic duct.

EUS-FNA may be beneficial for patients with pancreatic cystic lesions with the features listed in Table 3.^[5,15,21]^ In those patients, confirmation of negative cytology results and normal CEA levels in EUS-FNA may provide evidence to support the monitoring approach and deferral of surgical intervention.^[5,15,21]^ However, for most cases in Table 1, including ours, EUS-FNA were not used as auxiliary tools, with the exception of 2 cases^[5,22]^ and surgery was performed in all cases. If EUS-FNA were performed in the order of the algorithm and received regular follow-up, unnecessary surgery may have been avoided in our patients.

Knowledge of the most important and frequent characteristic radiologic findings of pancreatic cystic neoplasms may help radiologists make a diagnosis and reduce the possibility of unnecessary surgery.^[23]^ However, it should not be overlooked that sometimes there may be atypical findings, causing confusion about the diagnosis.

Although very rare, as in these cases, it is necessary to be aware that communication with the MPD can be seen even in the imaging findings of SCN mimicking IPMN. Therefore, particular attention should be paid to interpretation of clinicoradiologic findings of pancreatic cystic lesions, especially to the decision of surgical intervention.

## Author contributions

**Conceptualization:** Jisun Lee.

**Supervision:** Jisun Lee.

**Writing – original draft:** Mihyeon Park.

**Writing – review & editing:** Jisun Lee, Yook Kim, Kyung Sik Yi, Bum Sang Cho, Chi-Hoon Choi, Kil Sun Park.
